# Re-envisioning health promotion: Thinking and acting salutogenically towards equity for historically resilient communities

**DOI:** 10.1177/17579759211035089

**Published:** 2021-09-02

**Authors:** Fungisai Gwanzura Ottemöller, Tulani Francis L. Matenga, J. Hope Corbin, Humaira Nakhuda, Peter Delobelle, Christa Ayele, Nikita Boston-Fisher, Stephanie Leitch, Josette Wicker, Oliver Mweemba

**Affiliations:** 1 University of Bergen, Faculty of Psychology, Bergen, Norway; 2 University of Zambia, Lusaka, Zambia; 3 Western Washington University, Bellingham, Washington, USA; 4 IUHPE Student and Early Career Network, Toronto, Ontario, Canada; 5 University of Cape Town, South Africa; Vrije Universiteit Brussel, Brussels, Belgium; 6 IUHPE Student and Early Career Network, Philadelphia, Pennsylvania, USA; 7 McGill University, Montreal, Quebec, Canada; 8 IUHPE Trustee for Partnerships, NARO; University of Suffolk, United Kingdom; WOMANTRA, Trinidad and Tobago; 9 IUHPE Student and Early Career Network, Seattle, Washington, USA

**Keywords:** Health equity, salutogenesis, health promotion, resilient communities

## Abstract

This paper explores how the salutogenic theory can enable us to re-envision health promotion work with marginalized communities, towards an approach that acknowledges and honours their resilience. We use the three core concepts in Antonovsky’s salutogenic model of health – sense of coherence, generalized resistance resources and specific resistance resources – to explore the theory’s relevance to health equity, thus presenting new opportunities for how we might radically re-evaluate current health promotion approaches. We conclude that a more equitable health promotion requires increased participation of marginalized communities in shaping their futures and suggest a new model for historically grounded salutogenic health promotion.

## Introduction

Addressing health equity is a fundamental concern of the field of health promotion. The core action areas of health promotion aim to address inequity in health by influencing public policy, addressing environmental conditions, organizing communities, reorienting health services and developing personal skills (1). However, despite these goals, much of this work is funded and framed in ways that pathologize communities of colour, indigenous peoples and other marginalized groups. Discourses and interventions have been paternalistic in their approach and focused on ‘helping’ vulnerable communities, with an emphasis on ‘need’, and a deficit orientation.

Antonovsky (2) offered helpful critiques of health promotion’s persistent focus on pathology. His pioneering salutogenesic theory has provided the field with a framework for shifting thinking away from negative factors that cause disease^1^ towards positive factors that generate health. This approach is consistent with Morgan and Ziglio’s (3) health asset model where assets are ‘resources that individuals and communities have at their disposal, which protect against negative health outcomes and/or promote health status. These assets can be social, financial, physical, environmental or human resources’ (3, p.18). Significant work has been done to extend and examine the potential of a salutogenic orientation to promote health at the different socio-ecological levels (1).

In this paper, following calls for exploration of how we can further deconstruct hegemonic epistemologies (4), we argue that salutogenesis offers an important perspective in promoting health at the community level. By rejecting the unmerited pathologizing of traditionally oppressed communities as a starting point in our analysis, we allow ourselves the possibility to re-envision our work. We move to unearth or create approaches that acknowledge and honour the resilience demonstrated by ordinary actors (current and historic) in their pursuit of well-being. We activate our peers in the field to reprioritize health equity in meaningful ways. Antonovsky instrumentalized his theory in the Salutogenic Model of Health (SMH) and in this paper we use the model’s three core concepts (2) – sense of coherence (SoC), generalized resistance resources (GRRs) and specific resistance resources (SRRs) – to explore their relevance to discussions of health equity within diverse contexts and what lessons are most valuable for the transformation of current health promotion approaches.

## Sense of coherence (SoC)

As enshrined in the World Health Organization (WHO) Constitution (5), the highest attainable standard of health is the fundamental right of every human being. To improve the conditions in which people live, focus has been on mitigating the unfair, avoidable and remediable differences in health outcomes among vulnerable populations. This approach aims to be inclusive of disenfranchised groups and individuals, such as people of colour, Indigenous peoples, the LGBTQ+ community, people living with mental and physical disabilities and any person who is denied the chance to achieve their full potential. People, their health needs and overall experiences are fundamentally shaped by gender, race, class, sexuality, culture and citizenship, as well as by specific socio-political and historical structures (6,7). While these factors are recognized on some level, health promotion practice is still planned, funded and reported in deficit and ‘needs’-centred ways without the requisite attention being paid to underlying causes or equitable solutions. If we are going to reorient health promotion practice salutogenically and build on authentic empowerment, we must first recognize the strengths, resilience and solutions within communities in managing their own health. Such an orientation can build a sense of coherence through truth-telling about historical processes of harm and neglect, while highlighting uplifting stories of survival and resilience based on the actual experiences of the communities in question.

As Eriksson and Lindstrom (1) observe, health is created by complex relations between the individual and society and by an individual’s ability to identify and realize aspirations, as well as to satisfy needs and cope with their environment. SoC is a ‘global orientation to view life as structured, manageable and meaningful… which leads people to identify, benefit, use and re-use resources at their disposal’ (8, p.95). SoC is made up of three dimensions: comprehensibility, the ability to understand challenges faced; manageability, identifying the resources or assets to cope with these challenges; and meaningfulness, the motivation to engage with life’s challenges (9). The ability to manage stress in a globalized world characterized by rapid social and environmental changes is crucial for the maintenance and development of health. Below we use the three dimensions of SoC to interrogate whether current health promotion approaches are *relevant enough* for marginalized communities.

## Comprehensibility

A recognition of histories of oppression and structural inequality is central to understanding why marginalized communities continue to be disenfranchised and experience disproportionately negative health outcomes, in both mind and body. The trauma experienced by Black and Indigenous peoples, for example, is well documented. It is associated with the history of European colonization, which has stripped communities of their cultures, customs and language(s) – a deprivation that is further exacerbated by racism, state oppression and internal conflict (10,11). Historically, health promotion has had very little engagement with interrogating the lived experiences and realities of marginalized groups. The absence of this historical context leaves much unsaid about who bears responsibility for current inequities (12), and to what extent our current frameworks reinforce unequal power relations.

Spencer et al. (13) conducted a critical frame analysis to unpack the UN’s Sustainable Development Goals in relation to key health promotion indicators. This revealed a number of assumptions and ‘hidden’ value systems that codified hierarchy through language, and relied upon shared meanings of inequity (13). Key development terms such as (‘developed’ and ‘developing’) used to describe the economic status of countries, for example, are responsible for sustaining the primacy of Global North and other global powers. ‘Developing’ nations are positioned as recipients of action (i.e. those that need ‘developing’ or aid), while ‘developed’ nations and other powerful actors position themselves as having something positive and valuable to contribute (13). This systemic lack of recognition prompts us to ask, how can we enable marginalized communities to take control of their health without first examining the circumstances that have disadvantaged them?

Antonovsky argues that marginalized communities can often feel subjugated by hostile actors and the powers that be, causing them to experience ongoing stress as a direct result of lack of autonomy (14). As we seek to move the health promotion discipline forward, we must acknowledge the erasure and deception in our current narratives around health outcomes. Super *et al*. (15) state that the salutogenic model includes behavioural and perceptual mechanisms. The behavioural mechanism highlights the possibility to empower people through building their capacity to use their resources in stressful situations, while the perceptual mechanism implies that, for people to deal with everyday life stressors, they must be able to reflect on their understanding of stressful situations and identify available resources. They suggest that these interdependent empowerment and reflection processes may be relevant for health promotion activities that aim to strengthen SoC (15).

## Manageability

According to Antonovsky (2), manageability refers to the belief that we have the resources to cope with the stressors we face. The impacts of colonialism, heteropatriarchy and capital/GDP-focused development have not only systematically compromised the ability of marginalized communities to make sense of their circumstances but also robbed them of the crucial material resources needed to manage their day-to-day lives. Resources and people lost to colonialism and slavery, power and autonomy lost to debtors, unfair trade policies and laws, social and educational services eroded by structural adjustment programmes – all have trickled down through generations of history and to the people and communities who now occupy marginalized identities (13,16,17). These deprivations are central to the social determinants of health and are explored in later sections of this paper. Généreux and colleagues (18), in their paper on strengthening adaptive capacities of individuals and communities in times of pandemic, argue that community resources that are made available to help individuals deal with stressful situations are important to give voice to their personal experiences and share what they have learned to bring relational value. These narrative grounded insights, and others like these, can be used to inspire community-driven strategies to deal with stressors.

## Meaningfulness

Meaningfulness is considered the most important factor in determining a strong SoC: when a stressor or challenge is confronted and understood, and resources to cope are identified, what remains is whether there is the required motivation to engage with the process towards an achievable and satisfying end (2). As previously mentioned, health promotion and development are grounded in dominant discourses from the Global North. It is widely accepted that public health and medical interventions introduced by European colonizers and missionaries saved millions of lives in the Global South (19). Not enough attention has been paid to diseases brought in by colonialists, which in some regions wiped out Indigenous populations, or the lifestyles introduced that destroyed healthy Indigenous lifestyles. Moreover, the framing of health as the absence of disease has largely ignored indigenous and traditional ways of healing.

As we seek to move forward, how can we acknowledge erasure and deceptions in our current narratives of the past? How can we be realistic about the lack of and inappropriateness of resources, and collaboratively craft visions of the future that uplift and build on community resilience and Indigenous and subaltern ways of knowing, and centres bodily autonomy and planetary well-being?

## Generalized resistance resources (GRRs)

GRRs are ‘the characteristics of a person, a group, or community that facilitate the individual’s abilities to cope effectively with stressors and contribute to the individual’s sense of coherence’. These resources can be linguistic, ‘material, knowledge and intelligence, ego identity, coping strategies, social support, commitment and cohesion with one’s cultural roots, cultural stability, religion and philosophy’ (20, p.57). The quantity and quality of GRRs an individual is able to access has a direct impact on the development of their SoC, and thus their quality of life. Antonovsky (21, p.9) referred to GRRs as ‘phenomena that provide one with sets of life experiences characterized by consistency, participation in shaping outcomes and an underload–overload balance’. These life experiences contribute to the development of SoC. Consistency refers to the order and structure in one’s environment and provides the basis for comprehensibility; load balance is related to the balance between the resources available and the demands faced and is the basis for manageability; and participation in shaping outcomes refers to autonomy and control over one’s life and is the foundation for meaningfulness (20).

There are various ways in which GRRs can be contextualized and it is significant to note that how these resources are presented to an individual influences the meaningfulness of their experience and subsequently shapes outcomes (22). Mittelmark *et al*. (23) refer to an illustration by Bengt Lindstrom, depicting an individual traveling across the ‘river of life’ with a backpack full of GRRs that have been gathered over time. They explain that GRRs are then readily available for an individual to engage when needed, to manage tension and avoid stress (14). Examples of GRRs are found in descriptions of social capital and community resilience, in the disaster relief and management literature (see e.g. (24–26)), and social science and community psychology (see e.g. (25,27)).

Examples of social capital as a community GRR are found in studies documenting empowerment processes in communities and neighbourhoods (28), Aboriginal youth health (29) and the development of Community Action Networks under COVID-19-induced lockdown restrictions in South Africa (30). Social capital has been defined as the ability to secure benefits through membership of networks and other social structures (31). This definition distinguishes two components: a relational element connected to the social organizations of which the individual is a member, and a material component related to the resources accessible to the individual through group memberships (32). Hawe and Shiell (32) suggest that social capital’s political aspects may have been underrecognized, and need to be positioned in relation to other concepts such as sense of community and capacity-building. The concept has, however, gained much traction and is now firmly embedded in the health promotion literature. Sagy and Mana (33) define ‘sense of community coherence’ as the tendency of individuals to perceive their community as comprehensible, meaningful and manageable. They refer to reported positive relationships between a strong sense of community coherence and levels of resilience to stressful events. In their work with Palestinian Muslims and Christians in Israel, they examined the interplay between sense of coherence and inter-religious relations, showing how sense of community coherence is related to the perception of shared narratives of collective group history (33).

Idan et al. (20, p.57) listed ‘knowledge, commitment and cohesion with one’s cultural roots and cultural stability’ as GRRs. Knowledge gained through education is an important GRR that helps build and shape communities and their environments. Unlike traditional education which kept young people embedded in their communities (34), institutionalized education was used by colonial powers as an instrument of domination, oppression, subjugation and exploitation (35). It aided the reproduction of Western ways of knowing at the expense of traditional and indigenous knowledge, depriving communities of the GRRs they needed to help them cope and thrive in these newly constructed and alien environments (34,36).

Mavhunga (34) argues that traditional African education was integrative and educated the mind, body and spirit (10). Western education has disrupted local systems, leaving young people to be educated outside of their cultures and communities. Nwalutu (10) contends that post-colonial education systems remain as relics of colonialism and advocates for an ‘urgent … shift towards locally planned and executed educational programs and policies that are based on the people’s socio-cultural, environmental and experiential realities’. Mavhunga (34, p.451) provides an example from the Zimbabwean/African context and suggests a curriculum based on ‘the philosophy of unhu/ubuntu, rooted in African culture, characterised by qualities such as ‘responsibility, honesty, justice, trustworthiness, hard work, integrity, a cooperative spirit, solidarity, devotion to family and the welfare of the community’. These examples of GRRs are intrinsically embedded in the contexts within which people can facilitate the creation of enabling environments that build strong SoC and lead to improved health and well-being. Despite the less-than-ideal circumstances many marginalized communities experience, there are still examples of resilience and thriving that need to be amplified and further explored with the same scientific rigor as other examples cited and studied within the Global North.

## Specific resistance resources

Mittelmark et al. (23) underline the importance of differentiating between two concepts from the SMH, generalized resistance resources (GRRs) and specific resistance resources (SRRs). GRRs refer to inherent characteristics in an individual (or group) whereas SRRs are resources outside the individual (or group) that can be utilized to help cope with challenges. SRRs are situation specific and instrumental (23, p.71); they are ‘[…] optimised by societal action in which health promotion has a contributing role, for example, the provision of supportive social and physical environments’. Both GRRs and SRRs can be understood as health assets (see Morgan and Ziglio (3)) as they contribute to enabling individuals and communities to deal with challenges and to promote health and well-being.

Health, social welfare, education and political systems around the world are dominated by models developed in the Global North. Many of these models are neither comprehensible nor accessible to populations in large parts of the globe but are accepted as the gold standard because of the capitalist and neo-liberal models that dominate our international institutions. The Global North’s hegemony over what promotes health and well-being has long side-lined alternative knowledges (37). However, health models are emerging (38) to enable communities to access SRRs that are not only culturally appropriate but accessible and affordable.

Medical pluralism is supported by the WHO Traditional Medicine Strategy 2014–2023 (39), acknowledging the significant role traditional medicine plays in the Global South, and the widespread use and acceptance of complementary medicine in the Global North (39). This recognition of alternative ways of restoring and promoting health is crucial to support culturally relevant and accessible health-related SRRs. However, it is important to note the hierarchy in resource access, where certain SRRs, such as traditional medicine, are only considered acceptable when approved by institutions in the Global North. Previously, much of this knowledge was oral and handed down over generations, but countries like China and India have managed to promote alternative medicine in more integrated and institutionalized ways, with universities and colleges providing qualifications for holistic ways of treating mind, body and spirit (39). Traditional medical practitioners are appropriate SRRs for the contexts in which they are situated, and communities have the necessary GRRs to access them when needs arise.

Research on medical pluralism reveals that in many African countries, traditional healers still play an important role (40). Biomedical institutions are often not readily accessible and predominantly focus on somatic symptoms, and thus there is a need for SRRs congruent with local beliefs and traditions to help with spiritual or social stressors (40). Exercising medical pluralism indicates that these communities have high SoC and the necessary GRRs to access SRRs appropriate to their needs.

Unfortunately, in most contexts in the Global South, social welfare systems are largely based on models developed and implemented during colonial eras. In most traditional societies, communities looked after one another, for example by providing support for widows and orphaned children. The HIV/AIDS pandemic eroded traditional social support systems in many countries due to the premature deaths of many young adults. Botswana, for example, was hard hit by the pandemic, with up to 23% of children losing one or both parents (41). A local non-governmental organization (NGO) identified the need for these children to access psychosocial support, and developed a culturally relevant therapeutic method that is now implemented country-wide (41). Earth therapy enrols orphaned children from the same village in age cohorts in a 16-day wilderness-based therapeutic retreat and a follow-up programme for up to three years. The retreat uses rites of passage and rites of affirmation (similar to traditional initiation rites) to help cohorts build resilience, develop relationships and build community (42). The follow-up programme includes caregivers, community leaders and chiefs, social workers and police – providing a holistic support system (41). This is an example of how locally developed culturally appropriate mental health programmes are important SRRs for young people experiencing distress and can lead to positive sustainable outcomes.

Another example is the Office of Hawaiian Affairs’ (43, p.1) introduction of a Bill to address ‘the significant and pressing mental health needs of the Native Hawaiian community’. They recognized that Indigenous Hawaiians are disproportionally affected by mental health associated outcomes and risk factors (depression, abuse, suicide etc.), and yet they underutilize mental health facility SRRs (43). Based on evidence that facilities aligned with Indigenous Hawaiian cultural identity, values and beliefs promoted significantly better mental health outcomes, the authorities concluded that there is a need for facilities that are more compatible with Indigenous Hawaiians’ conceptualizations of illness, health and well-being (43).

Māori health models in New Zealand also offer a noteworthy alternative example. For instance, the *Te Pae Mahutonga* model (Southern Cross Star Constellation) incorporates four key tasks for health promotion: *Mauriora* (cultural identity), *Waiora* (physical environment), *Toiora* (healthy lifestyles) and *Te Oranga* (participation in society). They are situated within two key orientations for how the work should be done: with *Ngā Manukura* (community leadership) and *Te Mana Whakahaere* (autonomy) (44). This model, embraced by the New Zealand Health Ministry, is an example of a foundation from which truly supportive health services can be delivered.

The ability to use SRRs depends not only on their relevance to communities but also on how their repeated use is able to resolve challenges and create experiences that are meaningful (22). Current micro and macro systems need to be wary of creating passive relationships in how resources are used to achieve specific outcomes (22). As shown in the examples above, SRRs and GRRs designed and developed within a culture or community create an experience that is integrated with the perception and understanding of what is meaningful (22) and cannot always be produced or replicated by outsiders. For example, a pilot programme to address diabetes among South Asian Muslim women in Canada involving a physical activity intervention at a mosque revealed that participation was influenced by the provision of a convenient and accessible setting within a structured network that actively supported their religious and cultural needs (45). Active relationships thus become important to engage communities in how a resource can be developed and adapted (22). Strengthening the relevance and meaningfulness of SRRs strengthens the SoC of individuals and communities (22) and the way in which subsequent interactions between GRRs and SRRs are viewed as relevant and usable.

## Discussion

In 1996, Aaron Antonovsky proposed the salutogenic theory as a guide for health promotion (2); 25 years later, this paper attempts to outline a reassessment of health promotion’s health equity agenda. We draw on the interdisciplinary roots of the field and use a historical and decolonial lens to examine our limited engagement with the historical roots of health inequity. We argue for a more salutogenic orientation to health promotion and show how its key concepts – SoC (and its dimensions of comprehensibility, manageability and meaningfulness), GRRs and SRRs – can help us highlight the strengths of marginalized communities.

We outline the historical and systemic oppression of communities of colour, Indigenous peoples and other traditionally marginalized groups with the aim to honour these communities’ resistance to oppression and to highlight their resilience. Our discussion of SoC examines how the historical inequities founded on deliberate efforts to disenfranchise populations and erase their sense of personhood continue to impact these communities. For example, social justice movements like Black Lives Matter reveal that there is still much to be done, and that health promoters have an important role to play (46). We challenge the field to engage reflexively and critically on how, despite good intentions, our work is still embedded in neo-colonial agendas. We discuss how GRRs such as social capital and education can provide consistency and opportunities for participation that enable communities to make sense of their worlds, cope with challenges and build health and well-being. We highlight that SRRs such as culturally relevant projects and programmes developed within and with communities have a higher likelihood of success than those imposed from outside.

To move the health promotion discipline forward we must think salutogenically to help communities identify and activate health assets to shift the community engagement paradigm, amplifying and building on locally developed initiatives that work well.

**Figure 1.**
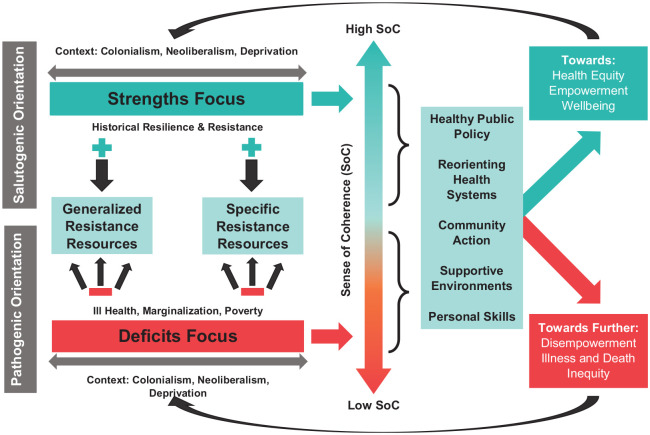
A salutogenic versus pathogenic approach to promoting health with historically resilient communities. Source: authors’ own elaboration.

Figure 1 below depicts the impact of a historically grounded salutogenic approach versus a pathogenic approach to health promotion. In both cases, health promotion is taking place within a historical context of colonialism, neoliberalism and intergenerational deprivation. A salutogenic orientation, which builds on historic and modern resilience and resistance, positively contributes to GRRs and SRRs and strengthens SoC. When this approach is implemented through the five action areas of health promotion, we move towards greater health equity, authentic empowerment, and well-being. The status quo is depicted as the pathogenic orientation, which is also situated in the historical context of colonialism, neoliberalism and intergenerational deprivation – but this approach frames communities in terms of deficits, which negatively impacts GRRs and SRRs and contributes to lower SoC. When this orientation is the basis of health promotion action, it results in further disempowerment, illness, death and inequity. Either approach feeds back and reinforces itself – the salutogenic orientation builds on itself positively, while the pathogenic orientation reproduces disempowerment and further fails to meet the needs of communities.

## Conclusion

Developing equitable programmes, systems and institutions requires that we openly acknowledge alternative ways of knowing, take the role of supplicant, ask communities to lead in matters that affect their lives, and advocate for a truly bottom-up and participatory approach. By acknowledging how we have overlooked the deep-rooted causes of health inequity we begin to shift the paradigm and build a radical health promotion that truly works towards equity for all.

## Notes

1. Antonovsky (2, p.14) considered the dichotomous classification health/illness to be inappropriate for health promotion and proposed a model where individuals move between the health and disease continuum during their lives.
